# Antihypertensive Drug Class Interactions and Risk for Incident Diabetes: A Nested Case–Control Study

**DOI:** 10.1161/JAHA.113.000125

**Published:** 2013-06-21

**Authors:** Rhonda M. Cooper‐DeHoff, Steven T. Bird, Gregory A. Nichols, Joseph A. Delaney, Almut G. Winterstein

**Affiliations:** 1 Department of Pharmacotherapy and Translational Research, College of Pharmacy, University of Florida, Gainesville, FL (R.M.C.D.H.); 2 Division of Cardiovascular Medicine, College of Medicine, University of Florida, Gainesville, FL (R.M.C.D.H.); 3 Department of Health and Human Services, Food and Drug Administration, Center for Drug Evaluation and Research, Office of Surveillance and Epidemiology, Department of Epidemiology, Silver Spring, MD (S.T.B.); 4 Department of Pharmaceutical Outcomes and Policy, College of Pharmacy, University of Florida, Gainesville, FL (S.T.B., J.A.D., A.G.W.); 5 Kaiser Permanente Center for Health Research, Portland, OR (G.A.N.); 6 Department of Epidemiology, University of Washington, Seattle, WA (J.A.D.); 7 Department of Epidemiology, College of Public Health and Health Professions and College of Medicine, University of Florida, Gainesville, FL (A.G.W.)

**Keywords:** β blockers, diabetes, diabetogenic, drug interactions, hypertension, RAS blockers, thiazide diuretics

## Abstract

**Background:**

We aimed to determine how single and combination antihypertensive therapy alters risk for diabetes mellitus (DM).Thiazide diuretics (TD), β blockers (BB), and renin–angiotensin system blockers (RASB) impact DM risk while calcium channel blockers (CCB) are neutral. DM risk associated with combinations is unclear.

**Methods and Results:**

We enrolled nondiabetic patients from Kaiser Permanente Northwest with a fasting plasma glucose (FPG) <126 mg/dL between 1997 and 2010. DM cases were defined by a FPG ≥126 mg/dL, random plasma glucose ≥200 mg/dL, HbA_1c_ ≥7.0%, or new DM prescription (index date). We used incidence density sampling to match 10 controls per case on the date of follow‐up glucose test (to reduce detection bias), in addition to age and date of cohort entry. Exposure to antihypertensive class was assessed during the 30 days prior to index date. Our cohort contained 134 967 patients and had 412 604 glucose tests eligible for matching. A total of 9097 DM cases were matched to 90 495 controls (median age 51 years). Exposure to TD (OR 1.54, 95% CI 1.41 to 1.68) or BB (OR 1.19, 95% CI 1.11 to 1.28) was associated with an increased DM risk, while CCB and RASB exposure was not. TD+BB combination resulted in the fully combined diabetogenic risk of both agents (OR 1.99, 95% CI 1.80 to 2.20; interaction OR 1.09, 95% CI 0.97 to 1.22). In contrast, combination of RASB with either TD or BB showed significant negative interactions, resulting in a smaller DM risk than TD or BB monotherapy.

**Conclusions:**

Diabetogenic potential of combination therapy should be considered when prescribing antihypertensive therapy.

## Introduction

Type 2 diabetes mellitus (DM) is a major societal and clinical concern. In 2009, 655 000 adults were admitted to a hospital due to DM or related complications, and DM, which is a leading cause of cardiovascular (CV) morbidity and mortality worldwide, is associated with an estimated cost of $174 billion annually.^1–2^ These issues have forced consideration of controllable conditions that may predispose individuals to the development of diabetes.

Hypertension is a prevalent health challenge in the United States, affecting ≈30% of adults and 5% to 10% of adolescents.^3–5^ By 2025, it is anticipated to afflict 1.56 billion adults worldwide.^6^ Hypertension prevalence is closely linked with obesity, and both increase risk for DM.^4,7^ While lifestyle modification remains the first step in the hypertension treatment process, most individuals will require one or more antihypertensive drugs, which could influence blood glucose and DM risk.^8^ Use of antihypertensive drugs is common, and more than 472 million antihypertensive prescriptions were filled in US pharmacies in 2010.^9–10^ Some analyses have suggested that concurrent use of thiazide diuretics (TD) and β blockers (BB) can increase risk for DM, while renin–angiotensin system blockers (RASB), including angiotensin‐converting enzyme (ACE) inhibitors and angiotensin receptor blockers (ARBs), have been shown to reduce DM risk.^11–13^

Little is known about whether, and to what extent, coprescribing drugs from different antihypertensive classes can result in drug–drug interactions that might alter the glucose modifying effects of individual agents. Such drug–drug interactions could increase or decrease the DM risk, having potential additive or multiplicative effects. Understanding the effects of combination antihypertensive medication regimens on DM development is important, given the increasing number of individuals who require combination therapy.^14^ Therefore, our study objective was to assess risk for incident DM with combination antihypertensive therapy.

## Methods

Kaiser Permanente Northwest (KPNW) is a group‐model health maintenance organization that provides integrated health care to ≈475 000 members in the Portland, Oregon area.^15^ KPNW maintains electronic medical records (EMR) to document clinical interactions between physicians and patients. The EMR contains information on all inpatient and outpatient encounters, pharmacy dispensing data, and laboratory tests. Diagnoses are coded in International Classification of Disease—9th Revision—Clinical Modification (ICD‐9‐CM) format, while pharmacy data are recorded as national drug code, prescription order date, and days' supply. Height, weight, smoking status, and BP are continuously collected during routine physician care. To complete each patient visit, the clinician is required to enter between 1 and 20 diagnoses in the EMR. The KPNW organization provides online medical guidelines to assist clinicians in patient management for most conditions, including hypertension. These guidelines also recommend lipid screening for men aged ≥35 years and women aged ≥45 years. Fasting plasma glucose (FPG) tests are routinely ordered in conjunction with these lipid screening panels.

### Design

Despite attempts to standardized glucose assessment (obtained at the time of lipid assessment), physician suspicion for DM could lead to more glucose testing and DM diagnoses in high‐risk or health‐service‐seeking individuals (detection bias). To account for this, and to capture a population undergoing active monitoring for DM risk, we used glucose tests to develop a nested case–control study. Included patients were between the ages of 35 and 65, were enrolled in KPNW for least 18 months between January 1997 and December 2010, and had both prescription and medical coverage. Patients entered the study cohort at the first negative FPG test (<126 mg/dL) following a 6‐month look‐back period without evidence for manifest diabetes (based on medications, laboratory tests, and in‐ or outpatient visits with a diabetes diagnosis [ICD9‐CM 250.x]). Patients were required to have 1 year of plan eligibility after cohort entry. Patients were censored at development of DM, cessation of KPNW enrollment, or December 2010, whichever came first. This research was reviewed and approved by the institutional review and privacy boards at the University of Florida and KPNW.

### Diabetes Cases

For the purposes of this analysis, incident DM was defined as a new FPG ≥126 mg/dL, random plasma glucose (RPG) ≥200 mg/dL, an HbA_1c_ ≥7.0%, or a new DM prescription, where the index date of the cases was the first of these occur. Because guidelines for use of HbA_1c_ ≥6.5% as a DM diagnostic criterion were not in place in the United States until early in 2010,^16^ we used a cutoff of 7% in our study to better reflect diagnostic practice during the study period. We also performed sensitivity analyses whereby DM cases were restricted to those with at least one subsequent positive DM test within 1 year after original DM diagnosis. This test could include any of the following: a second FPG ≥126 mg/dL, RPG ≥200 mg/dL, HbA_1c_ ≥7.0%, or use of DM medications.

### Matched Controls

To account for a potential DM diagnostic bias during follow‐up, the pool of eligible controls consisted of patients with negative DM tests (values below the diagnostic threshold), where the index date for controls was the date of the negative DM test. Incidence‐density sampling was used to select 10 controls per case, matching on date of the DM test (±6 weeks), age at DM test (±5 years) and date of cohort entry (±6 weeks).

### Drug Exposure

The primary exposures of interest were the dispensing of drug(s) within the BB, TD, RASB, and CCB antihypertensive classes. Exposure was defined as an active days' supply in the 30 days prior to index date. Prescriptions filled at index date were excluded.

### Statistical Analysis

We used conditional logistic regression analysis to compute ORs and 95% CIs to evaluate associations between incident DM and drug exposure. We estimated both the main effects and the interactions between antihypertensive drug classes using the same statistical model. The drug–drug interaction term quantifies the excess (or reduced) risk beyond what would have been predicted from the combination of the individual effects of each drug. ORs for dual‐antihypertensive therapy were calculated using the log odds of the parameter estimates and interaction terms and the covariate matrix (see Appendix S1 for equation). Dual therapy ORs represent the total risk of the drug combination, which includes the effect of each drug individually as well as any excess (or reduced risk) due to the combination of drugs.

We considered as covariates gender, baseline age and FPG, as well as smoking status, lipid levels (including HDL, LDL, and triglycerides), systolic and diastolic blood pressure (BP), body mass index (BMI) and CV disease history defined as in‐ or outpatient visit with diagnosis of cerebrovascular disease (ICD9‐CM 430‐438), myocardial infarction (ICD9‐CM 410, 412), congestive heart failure (ICD9‐CM 428), coronary artery disease (ICD9‐CM 414), or peripheral vascular disease (ICD9‐CM 441, 443.9, 785.4, V43.4), all of which were assessed during the 6 months preceding cohort entry. To account for other medication use that could affect DM manifestation, we also included drug covariates defined during the 30 days prior to index date, including oral corticosteroids (prednisone, dexamethasone, hydrocortisone, triamcinolone, methylprednisolone, or prednisolone); statins (fluvastatin, lovastatin, pravastatin, simvastatin, atorvastatin, or rosuvastatin); and atypical antipsychotics and antidepressants (selective serotonin reuptake inhibitors, selective norepinephrine reuptake inhibitors, or tricyclic antidepressants).

Multiple imputation was used to impute missing values. The percentages of missingness for covariates were: HDL 12.4%, LDL 15.8%, triglycerides 10.3%, blood pressure 5.4%, smoking status 53.9%, and BMI 12.2%. All analyses were performed using SAS version 9.2, and *P* values <0.05 were considered statistically significant.

## Results

**Figure 1.**
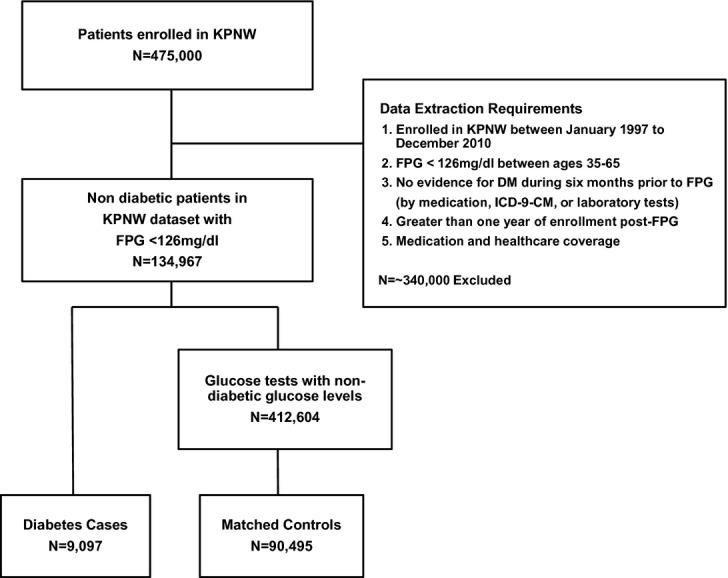
Flow diagram of Kaiser Permanente Northwest (KPNW) members included in the analysis. FPG indicates fasting plasma glucose; DM, diabetes mellitus; ICD‐9‐CM, International Classification of Disease—9th Revision—Clinical Modification.

Our cohort included 134 967 nondiabetic patients, with 412 604 glucose tests eligible for matching (Figure 1). From this cohort we identified 9097 patients who developed DM (cases), which were matched to 90 495 controls. Cases tended to be male with higher baseline FPG, BP, BMI, triglycerides and lower HDL, all characteristics consistent with an adverse metabolic phenotype (Table 1). Over 80% (7823) of cases were determined based on either FPG or RPG values. Of the 8207 DM cases with data available 1 year after DM diagnosis, we were able to confirm diagnosis in 6258 (69%) based on a second measured glucose value or HbA_1c_ or continued use of diabetes medication(s).

**Table 1.** Baseline Characteristics of Cases and Controls

	Cases	Controls
Patients, n	9097	90 495
Enrollment in KPNW system, y	4.5 (3.0)	4.5 (3.0)
Age, y		
Median (IQR)	51 (45 to 56)	51 (45 to 56)
35 to 45, n (%)	2531 (27.0)	24 447 (27.8)
45 to 55, n (%)	4102 (45.0)	40 742 (45.1)
55 to 65, n (%)	2464 (28.0)	25 306 (27.1)
Male%	55.2	44.8
FPG, mg/dL	104 (12.5)	96 (11.2)
Blood pressure, mm Hg		
Systolic	134 (16.5)	129 (16.8)
Diastolic	83 (9.8)	80 (10.0)
BMI, kg/m^2^	34 (7.4)	30 (7.1)
Lipid panel, mg/dL		
HDL	45 (12.8)	51 (12.2)
LDL	130 (36.9)	130 (38.0)
TRI	225 (224.4)	179 (179.3)
Ever smoke, n (%)	3548 (39.0)	34 841 (38.5)
Corticosteroid use*, n (%)	184 (2.0)	2423 (2.7)
Statin use*, n (%)	1791 (19.7)	20 377 (22.5)
CV disease*, n (%)	515 (5.7)	6028 (6.7)
Diabetes diagnosis		
FPG, n (%)	6523 (71.7)	
FPG, mg/dL	155 (52.0)	
RPG, n (%)	1300 (14.3)	
RPG, mg/dL	281 (115.0)	
New antidiabetic drug use, n (%)	655 (7.2)	
HbA_1c_%, n (%)	619 (6.8)	
HbA_1c_%	7.7 (1.1)	

Values are mean (standard deviation) unless otherwise indicated. KPNW indicates Kaiser Permanente Northwest; IQR, intraquartile range; FPG, fasting plasma glucose; BMI, body mass index; HDL, high‐density lipoprotein; LDL, low‐density lipoprotein; TRI, triglycerides; RPG, random plasma glucose; HbA_1c_, hemoglobin A_1c_.

* Corticosteroid use included any dispensed prescription for oral prednisone, dexamethasone, hydrocortisone, triamcinolone, methylprednisolone, or prednisolone; statin use included any dispensed prescription for pravastatin, lovastatin, simvastatin, atorvastatin, rosuvastatin, or fluvastatin.

* CV disease was defined as diagnosis of stroke, myocardial infarction, congestive heart failure, coronary artery disease, or peripheral vascular disease before cohort entry.

**Figure 2.**
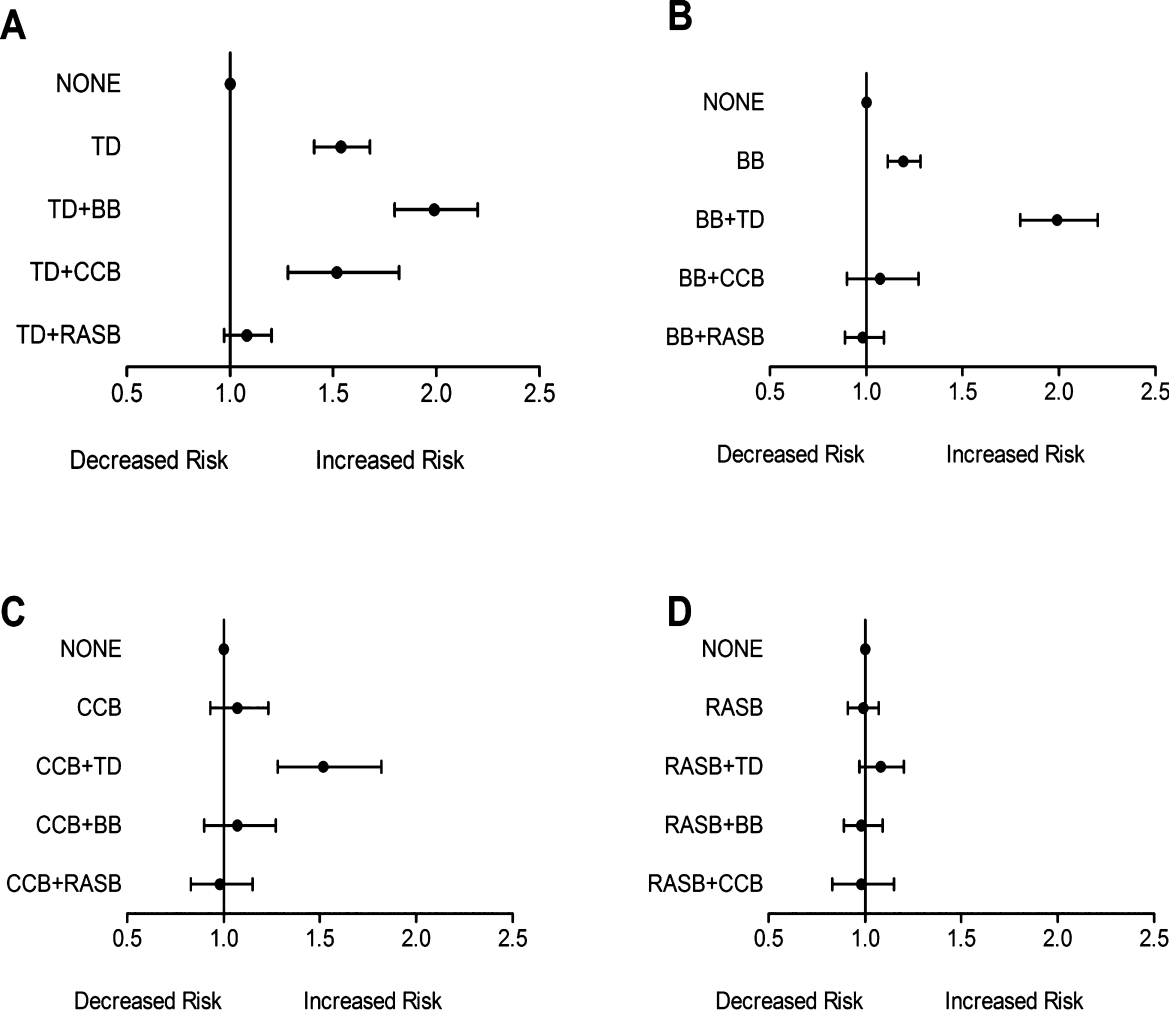
Adjusted odds ratio and 95% confidence interval for risk of diabetes among members in the Kaiser Permanente Northwest database who were prescribed (A) thiazide diuretics (TD), alone or in combination; (B) β blockers (BB), alone or in combination; (C) calcium channel blockers (CCB), alone or in combination; and (D) renin–angiotensin system blockers (RASB), alone or in combination. Members exposed to none of the drug classes constitute the reference group.

Drug exposure, by antihypertensive drug class or class combination, and risk for incident DM is summarized in Table 2 for cases and controls. Cases were more likely to be exposed to BB, TD, and RASB, overall and as part of a combination antihypertensive regimen. The top right quadrant of Table 2 describes the risk for DM based on exposure to each of the 4 drug classes individually. The lower right quadrant of Table 2 describes the risk for DM for each of the possible combinations of antihypertensive drug classes. There were no drug–drug interactions that significantly increased DM risk beyond the expected aggregate effect of the single drug classes. The combination of TD+RASB or BB+RASB resulted in significant interactions that had lower DM risk than would be observed from either the TD or BB alone. On the other hand, the combination of TD+BB and TD+CCB resulted in the fully combined diabetogenic risk of each agent, with adjusted ORs for DM of 1.99, 95% CI 1.80 to 2.20, and 1.52, 95% CI 1.28 to 1.82, respectively. A forest plot of the risk for DM for each drug class, individually and in combination with the other classes is shown in Figure 2.

**Table 2.** Individual and Combined Effects of the Antihypertensive Drug Classes on the Risk for Diabetes

Drug Use	Cases (n=9097)	Controls (n=90 495)	OR (95% CI)	
			Crude	Adjusted*
Individual				
None*	50.4	58.7	1.00	1.00
TD	21.5	14.3	1.64 (1.56 to 1.73)	1.54 (1.41 to 1.68)
BB	28.5	23.3	1.32 (1.26 to 1.38)	1.19 (1.11 to 1.28)
CCB	8.2	7.7	1.07 (0.98 to 1.15)	1.07 (0.93 to 1.23)
RASB	22.2	20.7	1.10 (1.04 to 1.16)	0.99 (0.91 to 1.07)
Combination			Adjusted Interaction Term**	Adjusted Effect for Class Combination
No combination*	77.1	81.7	1.00	1.00
TD+BB	10.1	5.7	1.09 (0.97 to 1.22)	1.99 (1.80 to 2.20)
TD+CCB	3.0	2.1	0.93 (0.78 to 1.11)	1.52 (1.28 to 1.82)
TD+RASB	8.3	6.6	0.71 (0.63 to 0.80)	1.08 (0.97 to 1.20)
BB+CCB	3.6	3.6	0.85 (0.71 to 1.00)	1.07 (0.90 to 1.27)
BB+RASB	9.7	9.0	0.84 (0.74 to 0.94)	0.98 (0.89 to 1.09)
RASB+CCB	3.6	3.7	0.93 (0.79 to 1.10)	0.98 (0.83 to 1.15)

Values are percentages unless otherwise indicated. TD indicates thiazide diuretics; BB, beta‐blockers; CCB, calcium channel blockers; RASB, renin–angiotensin system blockers; OR, odds ratio; CI, confidence interval; FPG, fasting plasma glucose; HDL, high‐density lipoprotein; LDL, low‐density lipoprotein; BP, blood pressure; BMI, body mass index; CV, cardiovascular.

* Adjusted for potential confounders including gender, baseline age and FPG, as well as smoking status, lipid levels, including HDL, LDL, and triglycerides, systolic and diastolic BP, BMI, glucose altering drug use (corticosteroids, antidepressants, antipsychotics, and statins) and CV disease.

* Patients who were exposed to none of the drug classes; these patients constituted the reference group for the individual drug analysis.

* Estimated excess or reduced risk of exposure to the combination of the 2 drug classes beyond the risk associated with exposure to each drug class individually (the risks of the individual drug classes appear in the top half of the table).

* Patients who were exposed to none of the drug combinations; these patients constituted the reference group for the combination drug analysis.

In a sensitivity analysis restricted to DM cases with a subsequent positive DM test, we observed similar results with slightly higher estimates for the diabetogenic risk of TD or BB (Table 3). We also observed similar DM risk for combination of TD+BB, OR 2.10, 95% CI 1.86 to 2.37, and TD+CCB, OR 1.62, 95% CI 1.31 to 2.01.

**Table 3.** Individual Effects of the Antihypertensive Drug Classes on the Risk for Diabetes in Patients With at Least 2 Tests Indicating Diagnosis of Diabetes

Drug Use	Cases (n=6258)	Controls (n=62 216)	OR (95% CI)	
			Crude	Adjusted*
Individual				
None*	49.4	59.2	1.00	1.00
TD	22.1	14.0	1.73 (1.63 to 1.85)	1.64 (1.47 to 1.83)
BB	28.9	23.0	1.36 (1.28 to 1.44)	1.26 (1.15 to 1.37)
CCB	7.8	7.7	1.02 (0.93 to 1.13)	1.02 (0.85 to 1.21)
RASB	22.9	20.5	1.15 (1.08 to 1.22)	1.05 (0.95 to 1.16)
Combination			Adjusted Interaction Term**	Adjusted Effect for Class Combination
No combination*	76.5	81.9	1.00	1.00
TD+BB	10.2	5.7	1.02 (0.88 to 1.17)	2.10 (1.86 to 2.37)
TD+CCB	2.9	2.1	0.97 (0.78 to 1.21)	1.62 (1.31 to 2.01)
TD+RASB	8.5	6.5	0.66 (0.57 to 0.76)	1.13 (0.99 to 1.28)
BB+CCB	3.4	3.7	0.81 (0.65 to 0.99)	1.03 (0.83 to 1.27)
BB+RASB	9.8	8.9	0.80 (0.69 to 0.92)	1.05 (0.93 to 1.18)
RASB+CCB	3.4	3.7	0.93 (0.75 to 1.14)	0.99 (0.81 to 1.21)

Values are percentage unless otherwise indicated. TD indicates thiazide diuretics; BB, beta‐blockers; CCB, calcium channel blockers; RASB, renin–angiotensin system blockers; OR, odds ratio; CI, confidence interval; FPG, fasting plasma glucose; HDL, high‐density lipoprotein; LDL, low‐density lipoprotein; BP, blood pressure; BMI, body mass index; CV, cardiovascular.

* Adjusted for potential confounders including gender, baseline age and FPG, as well as smoking status, lipid levels, including HDL, LDL, and triglycerides, systolic and diastolic BP, BMI, glucose altering drug use (corticosteroids, antidepressants, antipsychotics and statins) and CV disease.

* Patients who were exposed to none of the drug classes; these patients constituted the reference group for the individual drug analysis.

* Estimated excess or reduced risk of exposure to the combination of the 2 drug classes beyond the risk associated with exposure to each drug class individually (the risks of the individual drug classes appear in the top half of the table).

* Patients who were exposed to none of the drug combinations; these patients constituted the reference group for the combination drug analysis.

## Discussion

To our knowledge, this is the first large, population‐based study (over 130 000 individuals and over 410 000 available glucose tests) to investigate associations between different combinations of antihypertensive drugs on risk for DM. Our results indicate that treatment of hypertension with the combination of TD+BB was associated with significantly increased risk for DM, suggesting caution should be exercised when prescribing TD+BB combination therapy in individuals at risk for DM. Additionally, we found that interactions for combinations of TD or BB with a RASB were negative with regard to risk for DM, suggesting RASB are beneficial second line agents in those treated with a BB or TD. Importantly, these results remained consistent after sensitivity analysis.

A recent network meta‐analysis of hypertension clinical trials ranked the association of antihypertensive agents with incident diabetes as lowest for RASB (ACE inhibitors and ARBs), followed by CCBs, which appear neutral, and highest for BB and TD.^13^ Our data from the KPNW population are consistent with the findings in this meta‐analysis of RCTs, suggesting that incidence of DM following exposure to antihypertensive medications is generalizable to a much broader, real‐life population.

The onset of alterations in glucose after exposure to TD or BB have been reported to occur within 9 weeks of treatment initiation,^17^ and to continue with ongoing exposure.^18^ The European Guidelines on cardiovascular disease prevention in clinical practice (Version 2012), for the first time explicitly state that BB and TD are not recommended in hypertensive patients with multiple metabolic risk factors because of an increased risk for incident DM. This recommendation is considered Class III (harmful), and is associated with the highest level of evidence (A), suggesting an increasing level of awareness and significance of antihypertensive‐related adverse metabolic effects.^19^ Our data, showing increased risk with the TD + BB combination, are consistent with these recent guidelines.

Treatment with RASBs, including ramipril^20^ and valsartan,^21^ has also been investigated with regard to DM prevention. While treatment with ramipril has been associated with a significant increase in regression to normoglycemia, it has not been shown to significantly reduce risk for DM in patients with metabolic syndrome.^20^ In patients with impaired fasting glucose, valsartan was associated with a significant 14% decreased risk for diabetes.^21^ While the importance of DM prevention continues to be stressed,^22^ and screening high‐risk populations is cost effective,^23^ DM prevention guidelines and reviews do not give consideration to raising awareness of pharmacotherapy with known diabetogenic risk, like TD and BB, or make recommendations for alteration of therapy where appropriate to reduce that risk. Our data found negative interactions in combination therapy including RASB and either BB or TD, suggesting these drugs may offset some DM risk with BB or TD monotherapy, and that patients treated TD+BB would benefit from routine DM screening.

Whether DM that develops as a result of exposure to drugs with dysmetabolic effects has the same adverse consequences as DM that develops from other etiologies has been the subject of much debate.^24^ Most recently, the Antihypertensive and Lipid Lowering Treatment to Prevent Heart Attack Trial (ALLHAT) study, which observed increased risk for incident DM in patients treated with chlorthalidone,^25^ did not observe excess morbidity or mortality associated with incident diabetes.^26^ However, follow‐up in the ALLHAT Extension Study may have been insufficient to adequately assess long‐term adverse outcomes, and was not set up to assess microvascular complications. It is known that the deadly, debilitating, and costly complications of DM do not appear immediately after disease onset. Complications typically emerge a decade or more later, and importantly, the duration and extent of hyperglycemia predict complications.^22^ Follow‐up in most clinical trials is insufficient to fully elucidate the long‐term complications that may arise and additional research in this area is warranted. In the meantime, in the absence of solid clinical trial evidence, caution seems warranted when equally effective therapeutic alternatives to BB+TD combinations exist.

Our study has several strengths. First, we used a clinical practice data set (KPNW), which is a large primary care database containing longitudinal data on patients' medical history, and thus we were able to adjust for several important potential confounders, including baseline glucose blood pressure, cholesterol, smoking status and CV disease. Second, drug exposure was time varying as a result of the risk set sampling method used to select controls. Finally, we did sensitivity analyses, which, overall, produced results consistent with those of the primary analysis.

There are also some limitations worthy of mention. First, the identification of diabetes may have been subject to some misclassification. We relied on a single positive glucose test to establish incident DM, which may not reflect permanent dysglycemia. However, our sensitivity analysis, expanding the diagnostic requirement to include a subsequent test, confirmed our results. Second, based on the nature of an observational study design, residual confounding by indication and disease severity may be present. There could also be confounding by contraindication (eg, a physician refraining from prescribing TDs to a patient at a high risk of developing diabetes), which could have biased the results toward the null. Third, black race and Hispanic ethnicity have been associated with increased risk for diabetes,^27^ however, data on race/ethnicity were not systematically collected and thus are not reliably available in the KPNW dataset. It is estimated that only a small percentage of the KPNW population are nonwhite, and thus our findings require replication in other race/ethnic groups. Although we had access to BMI, we did not have waist circumference data, which precluded our ability to determine the presence of metabolic syndrome. However, our study did replicate known associations between antihypertensive monotherapy and DM risk based on RCTs,^13^ and reassures the appropriateness in using the KPNW cohort to assess drug‐induced DM, after accounting for available clinical characteristics. Fourthly, use of pharmacy dispensing data to define drug exposure does not allow for assessment of duration of drug exposure, nor does it guarantee patient adherence; however, any resulting bias from drug exposure misclassification would likely result in a bias toward reduced associations between drug exposure and DM onset. Finally, the state‐of‐the‐art EMR and clinical decision support within KPNW, in addition to focus on a privately insured and predominantly white population, may reduce study generalizability.

In conclusion, we found that antihypertensive regimens composed of either a TD or a BB were associated with increased risk for development of DM in the KPNW population. While the long‐term implications of drug‐associated diabetes are unclear, our observation that the DM risk for TD+BB combinations was stacked, with the full diabetogenic effect of each of the drugs being realized, suggests that TD+BB combination should be avoided in cases where alternative combination regimens with similar BP lowering efficacy are available. Conversely, treatment with a RASB in combination with a BB or TD resulted in drug–drug interactions that were negative, suggesting RASB containing combination may be preferred in those at increased risk for DM. Further research is needed to confirm our findings regarding association of antihypertensive combination therapy and DM risk.
